# Development of antimicrobial paper from unbleached bamboo ASAM pulps reinforced with nanofibrillated cellulose and chitosan

**DOI:** 10.1038/s41598-025-17210-y

**Published:** 2025-08-29

**Authors:** Areej Fathelrahman Abdallah, Ainun Zuriyati Mohamed, Areeba Siddiqui, Hina Khan, Paridah Md. Tahir, Mohammad Jawaid

**Affiliations:** 1 Institute of Tropical Forestry and Forest Products (INTROP), Universiti Putra Malaysia, 43400 Serdang, Selangor Malaysia; 2 Department of Forest Products and Industries, Faculty of Forestry, University of Khartoum, 13314 Shambat, North Khartoum, Sudan; 3 Department of Chemical and Petroleum Engineering, College of Engineering, United Arab Emirates University, P.O. Box 15551, Al Ain, United Arab Emirates; 4 Department of Wood and Fiber Industries, Faculty of Forestry and Environment, Universiti Putra Malaysia, 43400 Serdang, Selangor Malaysia

**Keywords:** Nanofibrillated cellulose, Chitosan, Mechanical properties, Barrier properties, Antimicrobial properties, Food packaging, Materials science, Nanoscience and technology

## Abstract

Despite being biodegradable, paper packages have restricted use in food packaging because of their strong tendency to absorb moisture and their high permeability to liquids and gasses from the environment. Consequently, investigating the application of biodegradable biopolymers, such as nanofibrillated cellulose and chitosan, to enhance characteristics is a pertinent technique. This study developed paper from unbleached bamboo alkaline sulfite anthraquinone and methanol (ASAM) pulps by incorporating nanofibrillated cellulose (NFC) and the antimicrobial agent chitosan (CS) into the papermaking process, offering a sustainable solution for advanced food packaging systems. The objective of this research is to investigated the potential impact of varying concentrations of NFC (5% and 10%) and CS (0.5%, 1%, 1.5%, and 2%) on the physical, mechanical, thermal, barrier, and antimicrobial properties of unbleached bamboo ASAM pulp, considering 4.000 and 6.000 beating revolutions to enhance the mechanical, thermal, barrier, and antimicrobial properties. The reinforcement of NFC and CS has significant enhancements to the paper’s properties. The results showed that incorporating 5% NFC and 1.5% chitosan at 6.000 beating revolutions has the optimum values of a tensile index and a burst index, where it reaches 85.16 Nm/g and 7.69 kPa m^2^/g, respectively. Besides that, it exhibited sufficient thermal stability to be used for food packaging applications, with the onset temperature of thermal degradation about 258.28 °C. The smoothness and porosity showed increases of 11.93% and 96.35%, respectively. This reflects a decrease in air permeability. Additionally, the paper sheets demonstrated antimicrobial activity against various food-borne microorganisms a notable rise of 43.96% and 49.75% against *Staphylococcus aureus* and *Candida albicans,* respectively. It concluded that the ASAM-reinforced paper with these great properties exhibited a promising prospect in food packaging applications.

## Introduction

The swift transformation of contemporary food consumption trends has presented considerable issues concerning food safety, quality, and sustainability. Overcoming these difficulties necessitates progress in agricultural productivity, nutrition, and food packaging technology^1^. The extensive usage of petroleum-based polymer food packaging has resulted in substantial environmental problems; thus, the creation of sustainable packaging materials is both crucial and urgent^2^. Furthermore, as worldwide concerns about environmental sustainability continue to grow, the packaging sector has been driven to discover novel solutions that not only meet the functional needs of the package but are also eco-friendly. In the quest to reduce plastic consumption, the paper industry has reemerged as a cutting-edge alternative^3^. Paper is an organic and environmentally degradable porous substance made from cellulose fibers that interact via hydrogen bonding. According to Tanpichai et al.^4^, this packaging material possesses the capability to accommodate a wide variety of products and exhibits desirable characteristics such as biodegradability, light weightiness, and non-toxicity. The permeable structure and properties of hydrophilic fibers of cellulose in paper or paperboard make them inadequate as barrier materials for food packaging. These materials exhibit low resistance to grease and water, are very permeable to gases and vapors of water, and are susceptible to microorganisms’ degradation^5^. The addition of additives to paper is minimal; it can significantly improve the paper’s performance^6^. Additives such as nanocellulose and chitosan have emerged as sustainable and abundant raw alternatives that are capable of improving the mechanical properties, barrier properties, and antimicrobial properties^6–8^.

Nanofibrillated cellulose (NFC) is a very promising nanomaterial derived from natural sources with dimensions of a diameter of ˂100 nm and a length that spans from a few hundred to thousands of nanometers obtained from natural cellulose fibers^9^. NFC is composed of amorphous and crystalline parts, and it may be produced by chemical and mechanical processes^10^. In food packaging, NCFs are widely used due to their abundant sources, remarkable barrier, and mechanical properties^11,12^. There are numerous explanations for the growing fascination with NFC as a novel category of paper constituents. This includes the fact that NFC possesses nanoscale lateral dimensions, which result in an expanded specific surface area. Additionally, NFC exhibits micrometer-scale lengths, a semi-crystalline structure comprised of elongated chains of cellulose, considerable intrinsic mechanical properties, commendable flexibility, a pronounced propensity for hydrogen bonding interactions with cellulosic fibers, and an inherent inclination to form intricate solid networks^13^. Furthermore, NFC is a natural material with high-performance processing capabilities. It is often used as a filler or additives to enhance the qualities of the matrix in active packaging materials. Currently, there is a growing integration of NFC into environmentally friendly and sustainable packaging materials. The abundance of hydroxyl groups on the surface of NFC allows for modification by a variety of methods and crosslinking with other biopolymers, such as chitosan. The purpose of this is to improve the antimicrobial properties of composite packaging^14^.

Chitosan is a commercially available amino polysaccharide that is derived from the natural biopolymer chitin by a process known as deacetylation. Chitin, as stated by Morin-Crini et al.^15^, is the predominant renewable polysaccharide found in the marine ecosystem and ranks as the second most prevalent polysaccharide found on Earth, behind cellulose. According to Liu et al.^16^, chitosan, a linear macromolecular polysaccharide, possesses several desirable characteristics, such as an exceptional capacity for film-forming and the added benefits of being renewable, non-toxic, biocompatible, and biodegradable. Chitosan is a compound composed of D-glucosamine and N-Acetyl-D-Glucosamine units that are connected by β (1,4) glycosidic linkages, resulting in a structurally intricate substance^17,18^. Additionally, the intrinsic antioxidant and antibacterial characteristics of chitosan allow the creation of active paper-based materials, which may be used to extend the shelf life of food and enhance its preservation^18^. Consequently, it exhibits considerable promise for use in food packaging applications. The incorporation of chitosan in the manufacturing of paper resulted in an improvement in its mechanical qualities. Furthermore, the utilization of chitosan and its derivatives has been found to augment the printing, electrical conductivity, barrier characteristics, and antibacterial attributes of paper, as demonstrated in research done by Egamberdiev et al.^19^ and Brodnjak^20^. The process of establishing hydrogen bonds between individual molecule chains, facilitated by the high crystallinity of the material, has garnered significant interest in the packaging industry and adhesive applications^21^. In addition, it has antimicrobial properties because the most frequently suggested antibacterial agent action of chitosan involves attaching to the anionic cell wall of bacteria, causing cell disruption, changing the permeability of the cell membrane, then attaching to deoxyribonucleic acid (DNA), preventing the process of the replication of (DNA), and ultimately killing the cell^22,23^. A significant amount of research has concentrated on the amalgamation of chitosan with nanocellulose to create composite films or coated paper for food packaging purposes. The manufacture of nanocellulose utilizing nanocellulose and nanochitosan as additives for the wet portion of paper greatly contributes to the advancement of antibacterial packaging technology derived from fiber sources and nanomaterials^24^. Costa et al.^7^ discovered that the fabrication of chitosan/CNC films exhibited improved characteristics, positioning them as sustainable alternatives for active food packaging. The chitosan/genipin/microfibrillated cellulose coating serves as a high-performance, environmentally sustainable substitute for fluorinated and non-biodegradable materials, with extensive applicability in grease-resistant food packaging systems^25^. The incorporation of nanocellulose onto chitosan film can enhance its antibacterial efficacy against *Escherichia coli*^26^.

However, more research needs to be conducted on the addition of chitosan and nanocellulose directly in unbleached pulp suspensions and their optimal concentration required for the enhanced strength of paper for food packaging applications. There are a few works that have addressed the addition of NFC and CS directly into pulp suspensions for improving paper’s properties in general^3,6,27^. As an addition to our previous research conducted by Abdallah and her group in 2023^28^, our current study looks closely at how chitosan strengthens nanofibrillated cellulose as advanced knowledge in this field and continues our previous research that focused on improving unbleached bamboo ASAM paper by adding nanofibrillated cellulose and chitosan. where our research is developing a novel paper specifically for food packaging applications by incorporating different loads of NFC and CS into an unbleached alkaline sulfite anthraquinone and methanol (ASAM) pulp suspension at 4.000 and 6.000 beating revolutions. The main aim was to assess the physical, mechanical, thermal, and barrier properties and antimicrobial characteristics of the modified paper sheet against food-contaminating microorganisms.

## Material and methods

### Material, chemicals and reagents

The bamboo culms (*Oxytenanthera abyssinica*) were collected from the Tolla forest in the Blue Nile State, Sudan. The culm nodes were removed, retaining just the internodes chipped into 25 mm in length. Following this, the chipped internodes were air-dried until they achieved a moisture content of approximately 10 ± 2%. The dried chips were enclosed in polyethylene bags before their transportation to the institute of tropical forestry and forest products (INTROP) at the University of Putra Malaysia in Selangor, Malaysia.

The chemicals and reagents used in this research were obtained from Sigma (Malaysia) and included sodium sulfite (Na_2_SO_3_- 98% pure), sodium hydroxide (NaOH- 99.9% pure), methanol (C_2_H_6_OH- with a purity of 99.9%), anthraquinone (C_4_H_8_O_2_- 97% pure), chitosan-medium molecular weight, (Sigma code-448877) (C_6_H_11_NO_4_- 75% pure), and aqueous acetic acid (CH_3_COOH- with a purity of 99–100%). Furthermore, the researchers procured commercially accessible nanofibrillated cellulose (NFC) obtained from the bleached pulp of oil palm empty fruit bunches (OPEFB) from ZOEPNANO Sdn. Bhd., located in Selangor, Malaysia. The reference antibiotics (Cephradine) used in this study were purchased from a medical company based in Sudan.

### Pulp preparation

The experimental conditions for ASAM pulping consisted of a fixed active alkali concentration of 17%, and a chemical ratio of Na_2_SO_3_/NaOH (70/30), with the addition of methanol (15%) and anthraquinone (0.1%). Furthermore, a ratio of 1 part liquor to 7 parts bamboo chips was chosen. The process of pulping was performed at a setting of 170 ºC for 90 min, at a pressure of 12.5 bar. Following the pulping process, the digester was gradually evacuated, and the pressure was alleviated via a vent in the digester. Afterward, the residual “black liquor” was drained, and bamboo pieces were collected. The collected chips were cleansed by scraping them with a wire under flowing tap water. The screen unit was cleaned repeatedly until the water’s color remained unchanged. Thereafter, the pulp was subjected to a Somerville screener. The papermaking process utilized only fibers capable of passing through a 0.15-mm screener plate, in compliance with T 275 sp-98^29^. The Unbleached ASAM screened pulp was pounded using a PFI mill (Paper and Fiber Research Institute). Pulp samples were subjected to beating at 4.000 and 6.000 revolutions per minute.

### Chitosan solution preparation

The solution was made by dissolving 1 g of chitosan (CS) in 200 mL of a 1% acetic acid solution, followed by stirring for 2 h at room temperature, as reported by Rahmaninia et al.^30^. The pH of chitosan solution was 4. According to Table 1, chitosan (CS) exhibits the following characteristics.

**Table 1:** The properties of chitosan.

Property	Value
Molecular weight	Medium molecular weight
Deacetylation	75–85%
Appearance (Color)	Off-white to beige and faint brown to light brown
Viscosity	200–800 cps

### Nanofibrillated cellulose preparation

The production of commercial nanofibrillated cellulose (NFC) derived from bleached oil palm-empty-fruit-bunch (OPEFB) pulp involves the utilization of a wet disc mill, commonly referred to as a high-shear ultra-fine friction grinder (ZOEPNANO Sdn. Bhd., Selangor, Malaysia). The NFC was stored in the cold room operated at 4 °C. Table 2 presents an overview of the attributes associated with nanofibrillated cellulose (NFC).

**Table 2:** The properties of nanofibrillated cellulose of oil palm-empty-fruit-bunch pulp.

Property	Value
Solid content	2%
Colour	White suspension
Diameter	8–20 nm
Viscosity	> 700

### Nanofibrillated cellulose (NFC) characterization

#### Transmission electron microscopy (TEM)

The suspension of NFC was achieved through the utilization of a high-resolution- transmission electron microscope (HR-TEM/JEOL Ltd.). The NFC suspensions underwent sonication via Ultrasonic processor/Sonifier, model (s00415938) with Frequency: 200 Hz manufactured by SONICS for 15 min after being diluted to a certain concentration of 0.01% (v/v). A drop amount of a diluted solution containing NFC and uranyl acetate was administered onto the grids coated with carbon and allowed to undergo staining for 5 min. The dispersion agent mentioned by Foster et al.^31^ facilitates the disintegration of NFC bundles and the preservation of individual nanofibrils. Subsequently, the produced sample was assessed by HR-TEM. Subsequently, the nanofiber sizes were manually measured using the image J analyzer tool based on the Hotaling et al.^32^ publication for Diameter J publication. The results obtained from the measurement of the diameters of 150 OPEFB nanofibers are shown as the average value of diameter for each set of data.

#### X-ray diffraction (XRD) analysis

The NFC content of the pulp derived from the oil palm-empty-fruit-bunch (OPEFB) was examined using of a Philips PW 3040/60 X’ pert pro-x-ray diffractometer, functioning at a wavelength of 1.5405980. The experimental conditions consisted of operational parameters set at 30 kV and 30 mA, with the instrument operating in step-scan mode. The step-scan mode covered a 2θ range from 5 to 40 degrees. The cellulose’s crystallinity index, or CRI, was estimated using the method proposed by Segal et al.^33^.

$${\text{Crystallinity index }}\left( {{\text{CRI}}} \right), \, \% \, = \, \left\{ {\left( {{\text{I}}_{{{2}00}} - {\text{I}}_{{{\text{am}}}} } \right)/{\text{ I}}_{{{2}00}} } \right\} \, \times {1}00$$

where I_200_ = The peak intensity associated with cellulose I and I_am_ = The peak intensity of the amorphous fraction.

#### Sheet preparation and testing

##### Preparation of incorporated paper

In order to produce a hand sheet with a density of 60 g/cm^2^, a pulp made from alkaline sulfite anthraquinone methanol was prepared. Chitosan was then added to the pulp at varying dosages of 0.5%, 1%, 1.5%, and 2% (according to the weight of the dried pulp). The addition of chitosan was carried out through disintegration using a disintegrator (model: Regmed-DSG-2000) at 1.000 revolutions per minute for 45 s. Subsequently, additional amounts of NFC at concentrations of 5% and 10% (according to the weight of the dried pulp) were included during a subsequent disintegration process, which involved 180.000 revolutions with Regmed (model: Regmed-DSG-2000) every time before its use, to facilitate NFC dispersion. High mixing energy is required to evenly distribute NFC in cellulose suspension. Consequently, they were integrated into the pulp before the disintegration phase, as prior studies indicated that the NFC were uniformly distributed throughout the pulp during disintegration^6,34^. The paper sheets were prepared utilizing a laboratory circle hand-sheet forming. Hand-sheet reinforcements were executed using one method (mixture method). The composite method involved the integration of NFC and CS with pulp during the paper formation process. Subsequently, an air-drying procedure was conducted in a controlled environment with a consistent temperature and humidity [(23 ± 1) °C and (50 ± 2) % relative humidity] for 24 h to get a dried ASAM-reinforced hand-sheet sample for additional experiments.

This study determined the concentrations of NFC with CS and the number of beating revolutions based on our earlier research^6,35^, which serves as a foundation for future experiments.

##### Properties of incorporated papers

The physical and mechanical tests of unbleached bamboo ASAM-incorporated paper were assessed using TAPPI standard procedures. The thickness of each incorporated paper sample was measured using an L&W micrometer in accordance with T 411 om–97^36^, specifies the thickness measurement for a physical test. While, the tensile and burst indexes for mechanical tests. The tensile index of incorporated paper was measured using a Buchel B.V. horizontal tensile tester, in accordance with T 494 om–01^37^. The measuring gap length of the test was established at 100 mm ± 1 mm. The dimensions of the sample were 150 mm in length and 15 mm in width. The test was conducted under controlled conditions of 23 °C ± 1 and 50% ± 2% relative humidity. The burst index of incorporated paper was measured by a Frank burst machine, in accordance with .T 403 om–97^38^. The samples with dimensions of 62 mm × 62 mm were prepared. The test was conducted under controlled conditions of 23 °C ± 1 and 50% ± 2% relative humidity. In addition, the test of air permeability was conducted on a smoothness and porosity tester utilizing the Bendtsen method as specified by ISO 5636–3^39^ 1992 and ISO 8791-2:2013^40^. The replicates of the testing were five hand sheets.

##### Thermogravimetric analysis (TGA)

Thermogravimetric analysis was used to investigate the thermal degradation characteristics of the samples by determining the reduction in weight as a result of increasing temperatures. The materials were subjected to TGA analysis utilizing a (Perkin Elmer Pyris series TGA 6 instrument located in Waltham, MA, USA). Prior to the application of heat, specimens weighing between 6 and 15 mg were introduced into an aluminum container. Subsequently, the specimen was exposed to a progressive temperature rise of 20 ºC per min in an environment containing nitrogen. The temperature range was selected to span from 30 to 1000 °C. The quantification of weight loss was determined through the utilization of a thermogravimetric analysis (TGA) curve, wherein a graphical representation of the percentage of weight loss was plotted against the corresponding temperature.

##### Biological activity testing

**Fig. 1:**
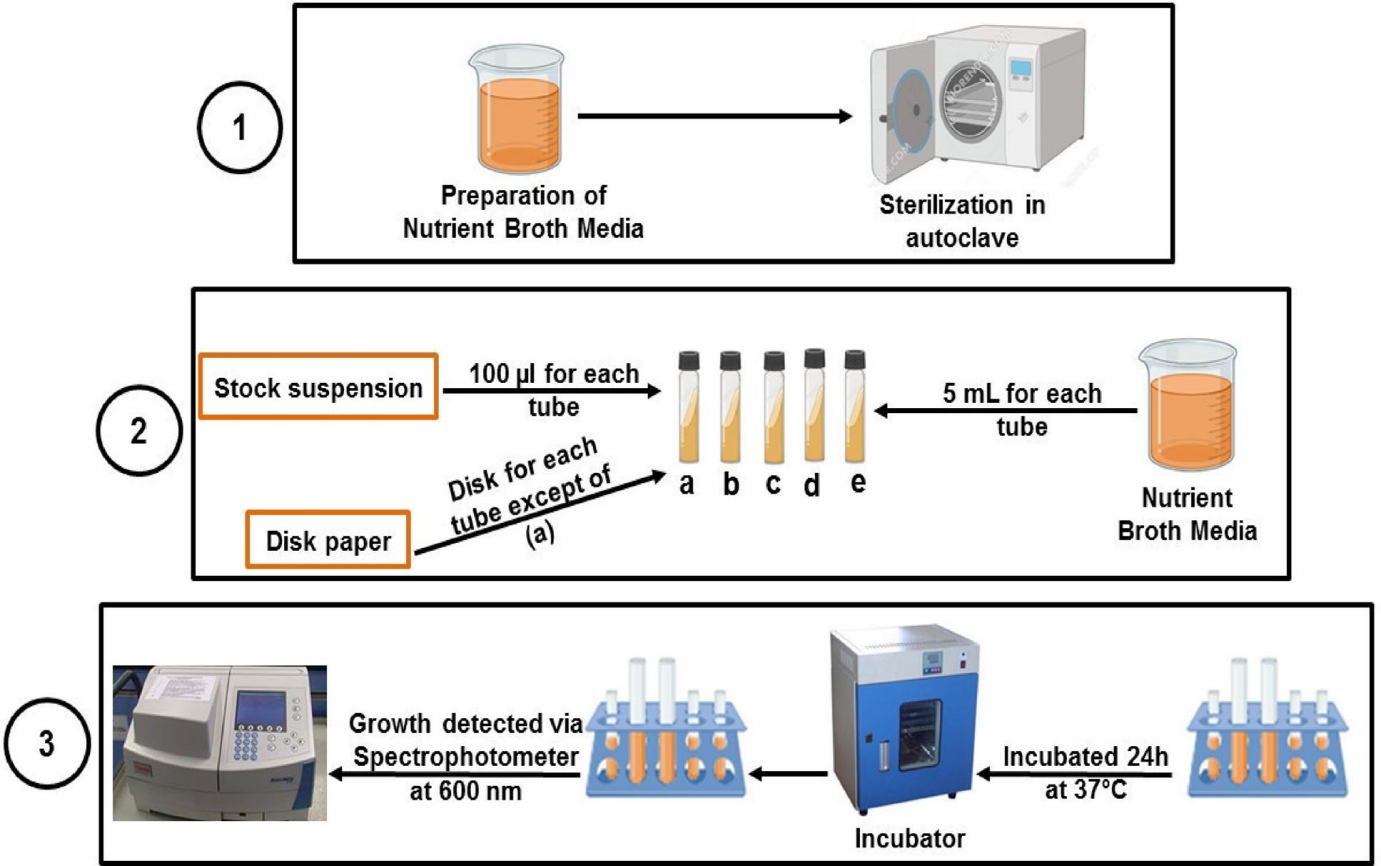
The steps for performing antimicrobial testing on NFC/CS-enhanced, unbleached ASAM paper. (**a**) Control (Stock suspension + Nutrient broth media); (**b**) *Staphylococcus aureus* (ATCC 43,300); (**c**) *Escherichia coli* (ATCC 25,922); (**d**) *Salmonella choleraesuis* (ATCC 10,708); (**e**) *Candida albicans* (ATCC 90,028).

The assessment of the antibacterial and antifungal characteristics of unbleached ASAM paper integrated with NFC/CS was conducted through the measurement of optical density (OD) at 600 nm for microbial growth. The material was divided into 6-mm-diameter holes using a paper puncher in aseptic conditions. Here’s a detailed explanation of the employed method: Firstly: The method involves preparing the nutrient broth medium and sterilizing the media and sample in an autoclave at 121 °C for 15 min. The steam under pressure autoclaves the nutrient broth medium and NFC/CH-reinforced ASAM disc paper. Steaming at a temperature higher than 100 °C is used in autoclaving. This is accomplished by utilizing high pressure. The autoclave is sealed and rendered airtight to facilitate pressure buildup, achieving 121 °C at a pressure of 15 lbs, which is maintained for a sterilization duration of 15 min. This approach eradicates spores^41^. Secondly, microbial cultures cultivated on nutrient agar slants were carefully transferred into 5 mL of sterile nutrient broth using aseptic techniques. The specimens underwent rigorous agitation and were thereafter placed in an incubator set at 37 ºC for 24 h. This particular stock was assigned to conduct research on antimicrobial agents. A volume of 5 mL of nutritional broth medium was filled into separate test tubes. A volume of 100 μL of microbial suspension and a paper disc containing the material under investigation was introduced into each tube. Subsequently, the tubes were incubated at a temperature of 37 ºC for 24 h. The optical density (OD) at 600 nm was utilized to measure the development of the microbial organism under investigation. The antimicrobial properties of the compounds under investigation were assessed against *Staphylococcus aureus* ATCC 43,300 (gram-positive bacteria), *Escherichia coli* ATCC 25,922, *Salmonella choleraesuis* ATCC 10,708 (gram-negative bacteria), and *Candida albicans* ATCC 90,028 (a yeast). The obtained experimental data were compared with the reference antibiotic (Cephradine). Figure 1 depicts the steps for conducting antimicrobial tests on NFC/CS-enhanced, unbleached ASAM paper.

### Statistical analysis

Analysis of variance (ANOVA) was used for data analysis using SAS software (version 9.4). The Duncan’s multiple range tests (DMRT) were employed to evaluate the significant difference among the obtained mean values.

## Results and discussion

### Nanofibrillated cellulose (NFC) characterization

#### Transmission electron microscopy (TEM)

**Fig. 2:**
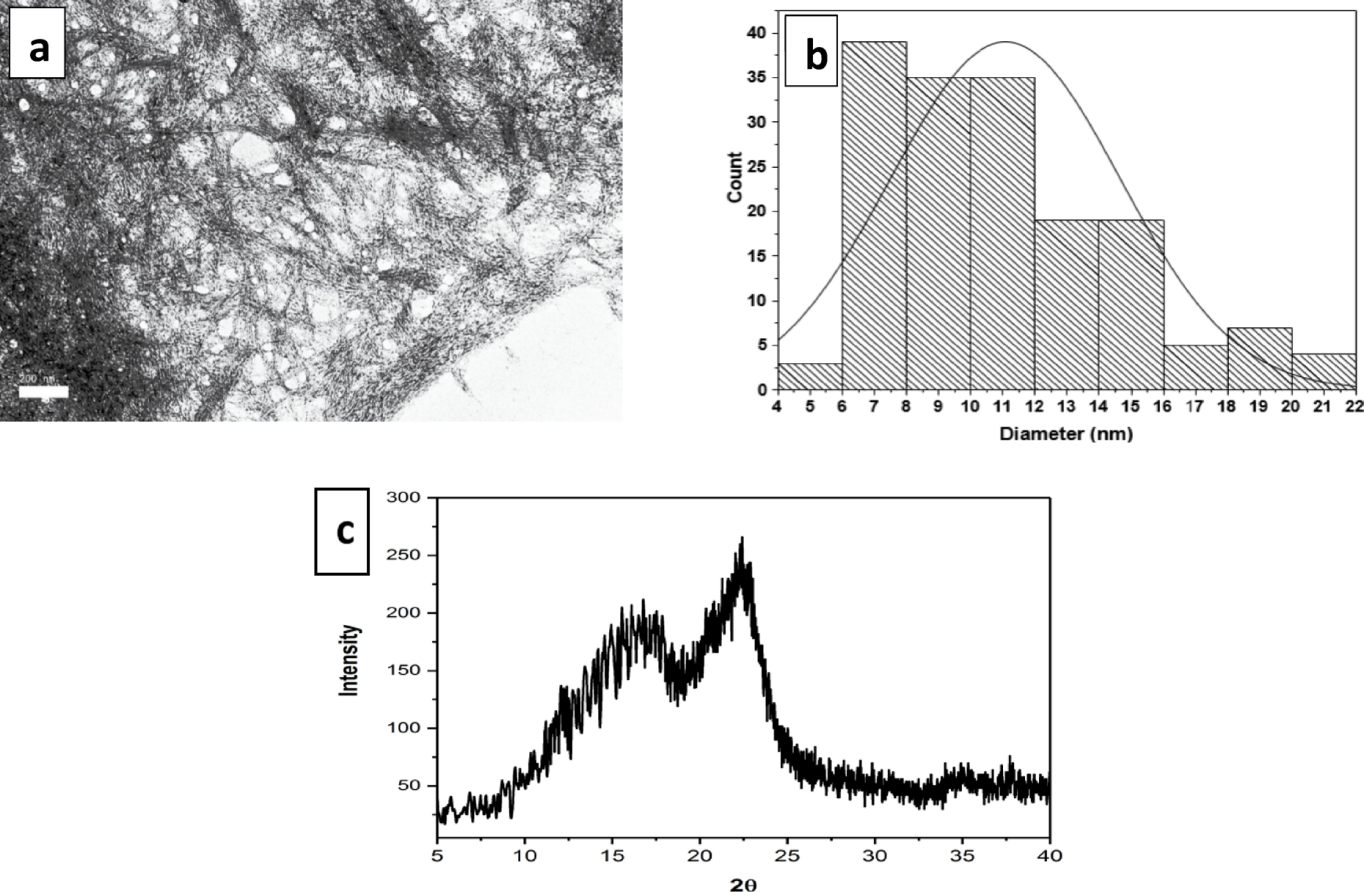
Nanofibrillated cellulose of oil palm-empty fruit-bunch pulp characteristics. (**a**)Transmission electron micrographs (scale bar 200 nm); (**b**) Distributions of diameter sizes. (**c**) Patterns of X-ray diffraction of NFC from bleached oil palm-empty fruit-bunch (OPEFB) pulp.

Figure 2a displays transmission electron microscope images of the NFC derived from bleached oil palm-empty-fruit-bunch pulp using either the ultra-fine friction grinder or wet disc mill. The TEM images depict nanofibers with sizes ranging between 5 and 21 nm, with an average diameter of 11.07 ± 3.59 nm, as shown in Fig. 2b. In TEM analysis at higher magnification, an aggregated structure of (OPEFB) NFC can be seen, which forms a highly entangled web-like cellulose nanofiber, demonstrating the drastic reduction of fiber size after mechanical treatment. As a result, in the course of this procedure, the cellulose slurry is moved between stationary and spinning grinding stones (disks), thereby preventing the occurrence of clogging. Furthermore, the cell wall undergoes delamination, and the nanofibrils experience individualization as a result of the shearing forces that arise between the discs. Moreover, the application of shearing forces several times led to the generation of significant energy, which therefore led to the formation of thinner fibrils. In comparison to previous studies on the extraction of nanofibrillated cellulose from various forms of agricultural waste, the present study yielded a diameter measurement of approximately 11.04 nm. This finding aligns closely with the outcomes obtained by^42^, who isolated nanofiber cellulose from sugar palm fibers by the process of high-pressure homogenization, resulting in a diameter of 11.54 nm. Conversely, the diameter of nanofibers obtained from cellulose derived from oil palm empty fiber bunches was found to be larger, measuring 32 nm, and was achieved through the use of a high shear ultrafine friction grinder^43^. In addition, Hongrattanavichit and Aht-Ong^44^ successfully obtained cellulose with a diameter ranging from 3 to 8 nm by isolating higher-than-nanofiber cellulose from sugarcane bagasse by the process of steam explosion and high-pressure homogenization techniques.

#### XRD analysis of NFC

X-ray diffraction (XRD) is a widely employed method for the analysis of the crystalline structure of lignocellulosic materials. The literature widely recognizes that the molecular composition of cellulose exhibits a combination of crystalline and amorphous regions^45^. This indicates that chains of cellulose will become bound together in the crystalline regions by mutual hydrogen bonding. Nevertheless, it has been observed that the non-crystalline sections of cellulose chains do not exhibit any hydrogen bonding^46^. Figure 2c depicts the pattern of x-ray diffraction of OPEFB-NFC. The crystallinity index of OPEFB-NFC was calculated as 55.31%. The results of this investigation indicate that the utilization of a wet disc mill (WDM) for mechanical treatment led to a notable enhancement in the crystallinity of the fibers compared to the crystalline index of 37% for powder from OPEFB fibers^47^. Figure 2c illustrates that the diffractogram of OPEFB-NFC exhibits an initial increase in peak intensity, subsequently followed by a fall and then a resurgence in intensity. The variations in intensity are attributed to the structural shift from amorphous to crystalline. The diffraction pattern of OPEFB-NFC showed a strong peak at 2θ = 22.4, which matches the typical peak values for the cellulose Iβ structure^48^. The findings of XRD analysis indicate the crystalline nature of nanofibrillated cellulose from OPEFB has potential as the low-cost feedstock. which beneficial in improving the reinforcing efficiency of NFC in polymer-based matrices, such as nanocomposites, for packaging applications^49^. 

#### Paper strength

In this experimental study, CS was introduced into the pulp at concentrations of 0.5%, 1%, 1.5%, and 2% as an antimicrobial agent. The pulp had undergone beating processes of 4.000 and 6.000 revolutions. In addition, each CS concentration was augmented with two levels of NFC at concentrations of 5% and 10% respectively, serving as a reinforcing agent. An investigation was done to examine the application of NFC/CS as a bonding agent in pulp and its potential as a replacement for the beating process.

#### Thickness

**Fig. 3:**
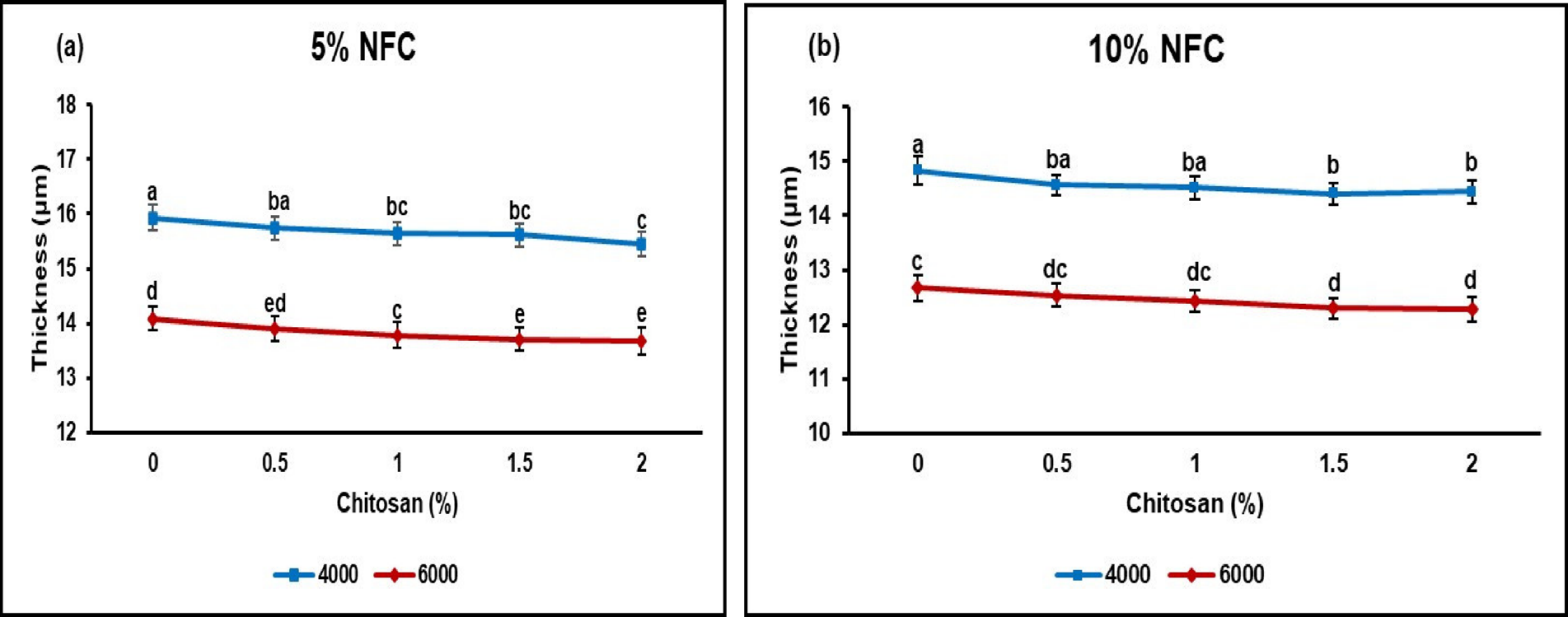
Thickness of paper incorporated with NFC/CS at different levels of beating revolutions (Means according to DMRT are substantially different at *p* ≤ 0.05 when followed by various letters).

The thickness measurements of the ASAM paper inclusion by NFC and CS at various beating revolutions are depicted in Fig. 3. It is evident that, as the NFC loading fraction increases, there is a significant decrease in the paper’s thickness. The findings align with existing scholarly literature, as previous studies have observed that increased beating revolutions lead to a reduction in paper thickness and bulk thickness, as measured by Ismail et al.^50^. Based on the research findings conducted by^51^ and^52^ , the incorporation of NFC-OX/NFC during the papermaking process leads to an increase in NFC content. This rise can be attributed to the filling of gaps between fibers, resulting in enhanced fiber interaction and the formation of an enhanced uniformity and denser paper structure. Consequently, paper of equivalent weight exhibits increased density and reduced thickness^53^. The addition of NFC (5 and 10%) to the suspensions of the pulp of bamboo ASAM papers at varying concentrations of chitosan (0, 0.5, 1, 1.5, and 2% o.d.) resulted in a range of thicknesses for the hand sheets produced, specifically ranging from 15.93 to 13.67 μm and 14.83 to 12.27 μm, respectively. The reduction in thickness observed in ASAM papers resulting from the inclusion of 5% NFC with varying concentrations of CS at different beating revolutions was somewhat more pronounced compared to the reduction observed in ASAM papers resulting from the inclusion of 10% NFC. The observed outcomes may be ascribed to an increased concentration of fillers in the raw materials, leading to a decrease in the ability of fibers to connect and resulting in a significant effect on the thickness of the paper sheets^54^

#### Tensile index

**Fig. 4:**
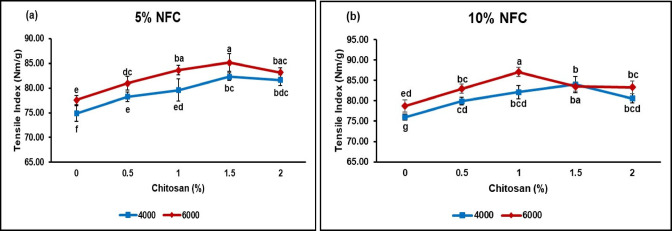
Tensile index of ASAM paper incorporated with NFC/CS at different levels of beating revolutions (Means according to DMRT are substantially different at *p* ≤ 0.05 when followed by various letters).

The specialized handling and transportation requirements of items need the consideration of essential qualities, for instance, elongation value and tensile strength at rupture in packaging materials^20^. The tensile index typically increased with higher levels of beating, as discussed in my previous study^28^. This study found that the tensile index values of unbleached ASAM paper without NFC and CS were 68.22 Nm/g at 4.000 revolutions and 75.13 Nm/g at 6.000 revolutions, respectively. In this research, we used these values as a baseline and discovered a significant improvement in the tensile index when we added NFC at 5% and 10% concentrations treated with different amounts of chitosan (0.5%, 1%, 1.5%, and 2%). The observed phenomenon can be ascribed to the enhancement of fiber contact facilitated by the NFC present in the slurry. This is achieved by effectively filling the interstitial spaces between fibers when the paper sheet is formed, hence leading to an increase in the bonding area^55^. Furthermore, the CS nanoparticle exhibited favorable performance in the treated samples owing to its distinctive characteristics. The chemical composition of chitosan bears a resemblance to cellulose, as it comprises hydroxyl and active amino groups. The aforementioned phenomenon facilitates enhanced cohesion between the fibers and fines, hence resulting in an increased bonding surface area of the paper sheet^56^. Figure 4 depicts the impact of CS inclusion on the tensile index of bamboo ASAM paper containing NFC at several degrees of beating.

In Fig. 4a, it can be observed that the inclusion of chitosan up to a concentration of 1.5% resulted in a rise in the tensile index. However, a decrease in the value of the tensile index was observed when higher concentrations of chitosan were added, regardless of the NFC % levels and beating speeds employed. We find that 1.5% chitosan gave the highest tensile index before it dropped at 2%. The observed phenomenon can be attributed to the thorough adsorption of chitosan onto the outer layer of the fiber when added at a concentration of 1.5%. That means the optimal dosage of chitosan in 5% NFC appears to be 1.5% at 6.000 beating revolutions, which is about 85.16 Nm/g. This finding is confirmed by Sarwarjahan et al.^57^, who discovered that the addition of chitosan to bamboo pulps resulted in nearly total adsorption onto the surface of cellulosic fibers, particularly on the surface of fine fibers at low doses. Furthermore, in this study, it was observed in Fig. 4b that the tensile index at 4.000 beating revolutions decreased after adding 1.5% CS, and it also decreased after adding 1% CS at 6.000 beating revolutions. The decrease is due to the pH range of pulp suspension during sheet formation being between 4.92 and 4.99. This is supported by the previous study done by Rohi et al.^58^, which found that the variation in data could be attributed to the influence of pH, which causes chitosan to transform into a more positively charged polyelectrolyte under acidic conditions. This alteration can affect the zeta potential of the fiber, causing it to exceed the zero point and resulting in a decline in formation due to increased flocculation.

#### Burst index

**Fig. 5:**
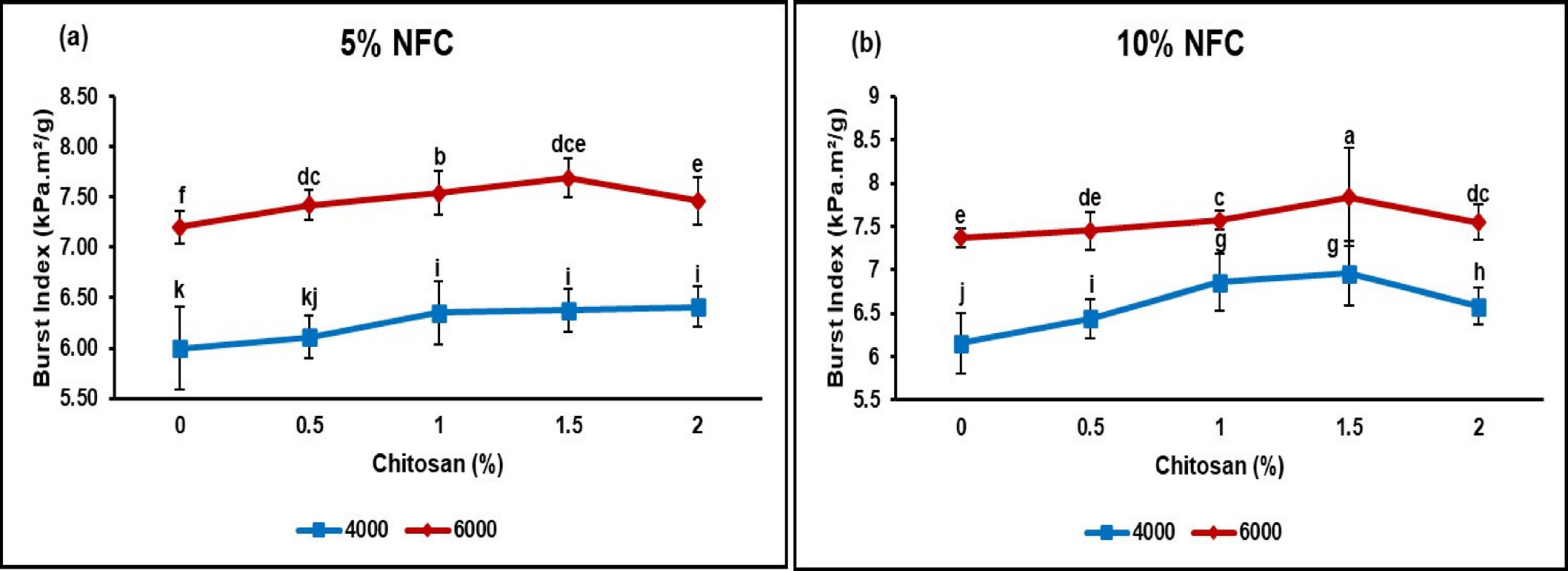
Burst index of ASAM papers incorporated with NFC/CS at different levels of beating revolutions (Means according to DMRT are substantially different at *p* ≤ 0.05 when followed by various letters).

The term “bursting strength” pertains to the capacity of the packaging material to preserve its contents. Consequently, it can be inferred that low-quality paper would have a propensity to rupture with relative ease^59^. The degree of fiber bonding in the papermaking process exhibits a strong correlation with burst strength. The earlier study on the burst index of ASAM pulp without additives revealed that the unbleached ASAM paper, devoid of NFC and CS, exhibited values of 5.84 and 7.10 kPa m^2^/g at 4.000 and 6.000 beating revolutions, respectively^28^. This research uses these values as a starting point and showed a significant improvement in the burst index when adding NFC at 5% and 10% levels, along with different amounts of chitosan (0.5%, 1%, 1.5%, and 2%). The term “burst” pertains to the maximum pressure that paper is capable of withstanding prior to experiencing rupture. Burst strength is influenced by inter-fiber bonding, fiber length, and sheet stretch, as stated by^60^. The burst index of the incorporation papers is depicted in Fig. 5.

Nanofibers often possess a significant specific surface area and engage in physical interactions with both fibers and other nanofibers. According to Kasmani and Samariha^61^, an augment in the number of hydrogen bonds results in a reduction in the space between fibers. The aforementioned aspects serve to enhance the interconnections among the internal fibers, resulting in heightened efficacy and stability of the fiber network. This is achieved by impeding the sliding of fibers, thereby bolstering the stability of the fiber network^62^. In contrast, the inclusion of CS has the potential to facilitate the infiltration of a similar chemical structure and fiber arrangement between chitosan and cellulose fibers, resulting in a high level of compatibility^22^. The inclusion of nanofibrillated cellulose (NFC) at concentrations of 5% and 10%, as depicted in Fig. 5, resulted in an augmentation of the burst index. The addition of 1.5% CS resulted in the most significant enhancements in both paper sheets at 6.000 and 4.000 revolutions, surpassing all other beating methods. However, when including paper sheets using 5% NFC at 4.000 revolutions, the highest burst index value was achieved with the addition of 2% CS. For instance, the highest increase in burst strength at 7.84 kPa m^2^/g was attained at an additional level of 10% NFC, 1.5% chitosan, and 6.000 beating revolutions. The potential advantage of using NFC to burst strength might be attributed to its substantial surface area resulting from its nanoscale dimensions. Furthermore, the bonding network was strengthened via the establishment of a robust hydrogen connection between fiber drying and NFC, as demonstrated by Latifah et al.^60^. The incorporation of NFC/CS into the bulk suspensions of ASAM unbleached pulp resulted in an elevation of the burst index value of the final sheets, similar to the observed enhancement in the tensile index. The various factors that influence the strength of paper, including the machine orientation, horizontal strength, and tensile length of the paper sheets, can have an impact on the burst index due to its close correlation with the bonding ratio and the forces of individual fibers^63^. The Burst index experiences an increase due to multiple factors, as previously addressed in the literature^30^. Based on the aforementioned research, it has been observed that cationic polymers, for instance, chitosan, possess the ability to effectively boost the internal bonding inside paper webs. This can be ascribed to the substantial concentration of cationic charges current in these polymers, particularly under acidic pH conditions. Consequently, the burst index exhibited an increase due to the augmented quantity of inter-fiber linkages resulting from the incorporation of NFC and CS. Based on the findings of physical and mechanical properties, nanofibrillated cellulose and chitosan-incorporated ASAM paper gave three good results at 5% NFC,1.5% CS-4.000, 5% NFC-1.5% CS-6.000, and 10% NFC-1.5% CS-4.000 beating revolutions (Table 3).

**Table 3:** Paper Properties of NFC/CH incorporated Paper.

Paper Properties	5% NFC–1.5% CS–4.000 Rev	5% NFC–1.5% CS–6.000 Rev	10% NFC–1.5% CS–4.000 Rev
Thickness (μm)	15.62	13.71	14.44
Tensile index (Nm/g)	82.29	85.16	83.99
Burst index (kPa m^2^/g)	6.38	7.69	6.96

NFC, Nanofibrillated cellulose; CS, Chitosan; Rev, Revolution.

The augmentation of paper properties using ANOVA analysis following NFC/CH enhancement is presented in Table 4. The results indicate that the addition of nanofibrillated cellulose and chitosan had a significant effect on the characteristics of the pulp and paper. The primary objective of NFC and CS is to occupy the spaces between the fibers and enhance the connection or adhesion of cellulose fibers, hence enhancing the cohesion of the fibers. NFC is an appealing strength-enhancing property for paper as a result of its high specific strength, high cohesive energy density, and high specific surface area, which are well-suited for enhancing the bonding between pulp fibers^64^. Similarly, chitosan possesses a cationic nature, the ability to form films, the capacity to connect, and a flexible structure. These characteristics allow the polymer to permeate the pores of the fiber and enhance the fiber crossings’ load-bearing capacity^30^. The unique requirements of handling and shipping products make the tensile strength of packaging materials crucial. In addition, the bursting strength of paper bags is crucial since it indicates the amount of pressure that paper can withstand before it ruptures^20^.

**Table 4:** An analysis of variance (ANOVA) of the effects of adding NFC/CH on paper properties.

NFC%	PFI Revolutions	df	*P*-value		
			Thickness index	Ten. index	Burst index
5%	4.000	4	0.0304*	0.0277*	0.0202*
	6.000	4	0.0366*	0.0291*	0.0240*
10%	4.000	4	0.0359*	0.0285*	0.0231*
	6.000	4	0.0381*	0.0298*	0.0253*

*Significantly different at *p* ≤ 0.05.

#### Thermogravimetric analysis (TGA)

The rapid process of thermal degradation of cellulosic materials at temperatures below 400 ºC is a widely recognized phenomenon^45^. The degradation of hemicelluloses, lignin, and cellulose is significantly influenced by the chemical differences that exist among them when exposed to varying temperatures. The initial stage of thermal degradation in lignocellulosic materials involves the breakdown of hemicelluloses, followed by lignin pyrolysis, cellulose depolymerization, active flame combustion, and oxidation of char^65^.

**Fig. 6:**
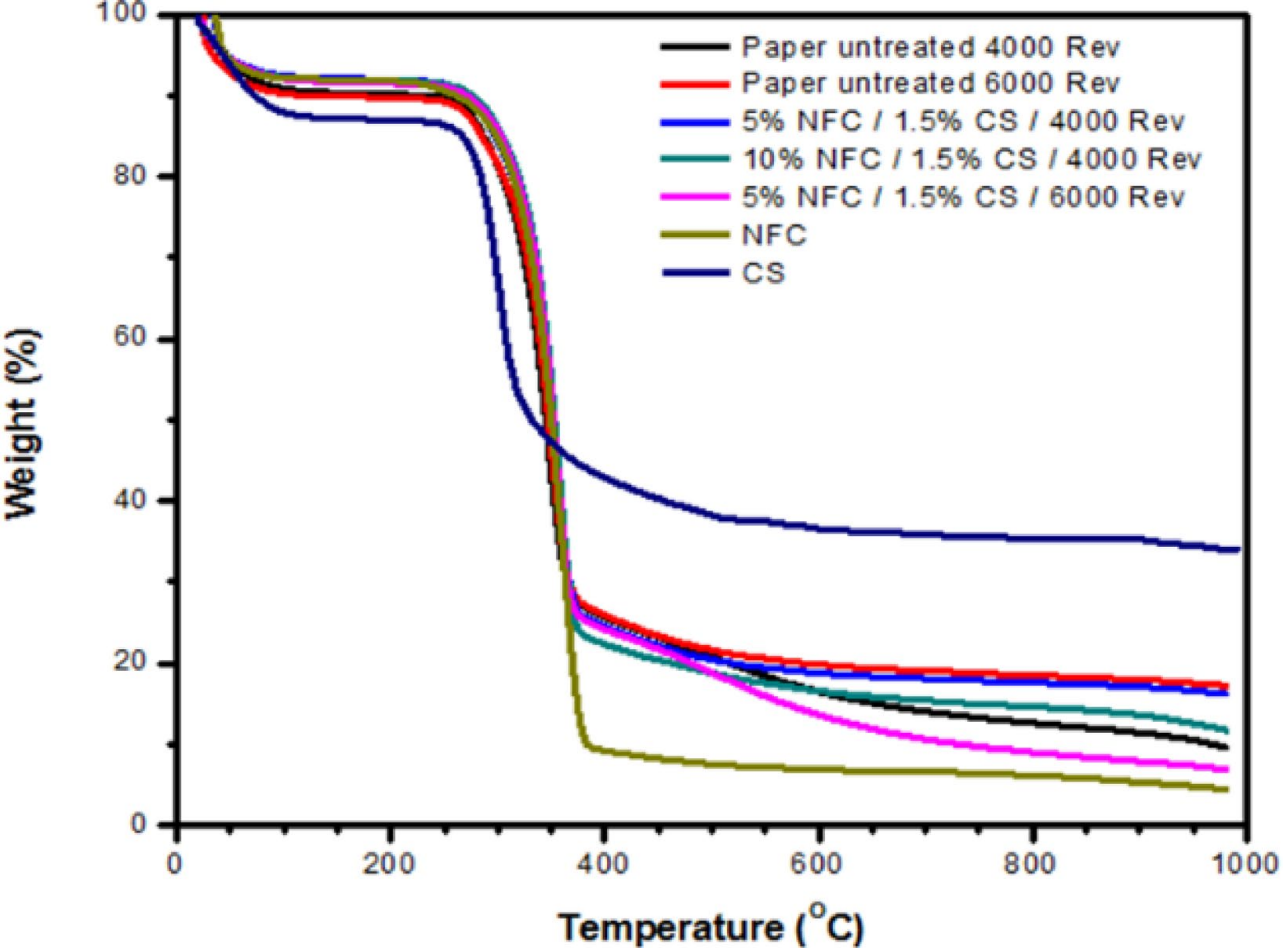
TGA thermograms of treated paper and untreated paper alongside commercial Nanofibrillated cellulose and chitosan powder.

The TGA analysis examined various samples, including untreated paper, treated paper, nanofibrillated cellulose (NFC), and chitosan (CS). Based on the thermograph illustrated in Fig. 6, it can be noticed that thermal degradation can be categorized into four distinct stages. The initial phase involves the reduction of fiber mass due to the physical adsorption and hydrolysis of fibers in the paper. The essence of the paper remains predominantly unaltered at this juncture^66^. All of the samples had a consistent pattern of degradation. It is evident that the disintegration of all samples commenced at a temperature of 30 ºC. Within the temperature range of 30 to 100 ºC, the initial weight of all fibers experiences fluctuations, amounting to approximately 6%, which can be ascribed to the process of converting absorbed moisture by evaporation, as seen by the depicted curves. The phenomena under consideration can be attributed to the presence of fibers and nanofibers that have the ability to evaporate absorbed water, as discussed by researchers^67,68^. The second decomposition temperature exhibited a value of around 252 ºC, however, the greatest decomposition temperature recorded was roughly 326 ºC. The observed temperatures corresponded to the disintegration of hemicellulose, amorphous cellulose, and the breaking of cellulose glycosidic bonds. The susceptibility of hemicellulose to thermal degradation can be ascribed to the existence of acetyl groups. The depolymerization of the lignin and crystalline cellulose areas occurred at temperatures exceeding 325 °C, reaching a plateau thereafter. Upon reaching the final temperature of degradation, the crystalline cellulose underwent total degradation.

The thermal stability of chitosan with a medium molecular weight is comparatively lower when compared to untreated paper, treated paper, and NFC. At temperatures beyond 400 °C, further disintegration of the chitosan takes place. The process of chitosan thermal decomposition involves the cleavage of glycosidic linkages amidst the N-acetylglucosamine and N-glucosamine domains, leading to the liberation of a diverse gas mixture comprising HO, NH_3_, CO_2_, CO, CH_3_COOH, and CH_4_^4^. Furthermore, Table 5 provides a comprehensive summary of the thermal properties of the samples, encompassing the initial breakdown temperature (T. _Onset_), the maximum degradation temperature (T. _Max_), and the proportion of residue at 1000 ºC.

**Table 5:** Temperatures of initial decomposition (T. _Onset_), maximum decomposition (T. _Max_), and percentage of sample residue as determined by the TGA curve.

Sample	Onset of Degradation (T. _Onset_) (ºC)	Maximum degradation (T. _Max_) (ºC)	Residue after Heating (%)
Paper untreated			
Paper at 4.000 Rev	237.32	312.69	9.60
Paper at 6.000 Rev	244.84	316.37	17.19
Paper treated			
5% NFC%/1.5% CS/4.000 Rev	253.47	320.37	16.18
5% NFC %/1.5% CS/6.000 Rev	258.28	325.03	6.85
10% NFC %/1.5% CS/4.000 Rev	258.90	324.27	4.42
NFC	264.21	326.43	11.65
Chitosan	246.83	281.26	37.20

Rev, Revolutions; NFC, Nanofibrillated cellulose; CS: Chitosan.

As a notably from the Table 5, The presence of biopolymers increased the onset temperature for thermal degradation. Where, various elements have the potential to influence thermal stability. One such aspect that has been identified as crucial is crystallinity^69^. An increased degree of crystallinity leads to cellulose domains that exhibit greater organization. According to Saurabh et al.^70^, a material that possesses a greater degree of structural organization and a higher concentration of cellulose molecules may demonstrate an increased strength of hydrogen bonding between adjacent cellulose chains. Consequently, this phenomenon can impede the process of thermal conduction through diffusion across the cellulose chains.

The first decomposition temperatures of treated paper samples with varying compositions were determined from the data provided in the table. Specifically, the values obtained for treated paper samples containing 5% NFC and 1.5% CS at 4.000 revolutions, 5% NFC and 1.5% CS at 6.000 revolutions, and 10% NFC and 1.5% CS at 4.000 revolutions were found to be 253.47, 258.28, and 258.90 °C, respectively. In contrast, the highest decomposition temperatures observed in the second thermal decomposition for the treated papers were 320.37 °C, 325.03 °C, and 324.27 °C. These temperatures were achieved under conditions of 5% NFC, 1.5% CS, and 6.000 revolutions and resulted in a higher thermal decomposition temperature of 325.03 °C compared with the other papers that were exposed to different treatments. Researchers have found that utilizing nanofibrillated cellulose, chitosan polymer, and beating processes in paper production has significant effects. The interaction between hydroxyl and amino groups in cellulose leads to the formation of CS, which can be influenced by the nitrogen content present in chitosan^71^. Additionally, using high-shear ultrafine friction while grinding can break down the non-crystal parts of cellulose, making the nanofibrillated cellulose more crystalline. This higher crystallinity contributes to improved heat resistance and enhanced thermal stability^72^. The beating process, as pointed out by Is. Ismail et al.^50^, mainly aims to strengthen the connections between cellulose fibers and boost their strength, which in turn enhances their thermal stability. Herein, the thermal stability of the samples was influenced by the addition of NFC and chitosan to the pulp, increasing the onset temperature for thermal degradation. This finding reflects a significant effect on the thermal stability of the samples. These samples can be used in food packaging applications based on the study of Wang et al.^73^, who reported that the materials intended for food packaging must withstand temperatures ranging from − 40 °C to 220 °C. Similar findings have been noted where adding nanocellulose and chitosan raises the temperature at which thermal degradation begins: unbleached NCF films^74^ and old corrugated container (OCC) paper treated with NC-DTPA-CS^66^.

#### Barrier properties

This study assessed the barrier characteristics by conducting tests on smoothness and porosity. Table 6 assesses the levels of smoothness and porosity exhibited by papers treated with alkaline sulfite anthraquinone methanol (ASAM), as well as the improvements achieved by incorporating nanofibrillated cellulose (NFC) and chitosan (CS) into ASAM sheets.

**Table 6:** Evaluation of smoothness and porosity of ASAM papers and incorporated ASAM paper with NFC and CS.

Sample	Smoothness (mL/min)	Porosity (mL/min)
Paper untreated		
Paper at 4.000 Rev	1755.00 (± 19.20)	378.30 (± 4.70)
Paper at 6.000 Rev	1850.80 (± 9.42)	255.00 (± 5.60)
Paper treated		
5% NFC %/1.5% CS/4.000 Rev	1705.00 (± 23.58)	14.00 (± 2.00)
5% NFC %/1.5% CS/6.000 Rev	1630.00 (± 18.62)	9.30 (± 2.70)
10% NFC %/1.5% CS/4.000 Rev	1680.90 (± 13.71)	10.30 (± 2.40)

Rev, Revolutions; NFC, Nanofibrillated cellulose; CS, Chitosan.

#### Smoothness and porosity

Paper is an extremely permeable material composed of a layer of intertwined fibers. Supplements have the potential to impact several characteristics of paper, such as its smoothness and ability to allow air to pass through^75^. In addition, paper smoothness refers to the degree of surface uniformity, which is impacted by how the paper is created or processed^50^. According to the data presented in Table 6, it can be evident that the level of smoothness was found to be higher when the beating revolutions were increased to 6.000 samples, as compared to 4.000 samples. Consequently, increased rotational speed yields smoother surfaces. The obtained outcome using a 5% concentration of NFC, 1.5% concentration of CS, and subjecting the mixture to 6.000 beating revolutions yielded a flow rate of just 1630.00 mL/min. This value is notably lower than the flow rate of the control sample, which measured 1850.80 mL/min.

The porosity of a paper is a measure of its capacity to absorb water. Furthermore, porosity is a fundamental characteristic employed to characterize the air-filled spaces existing between the fibers, as discussed by Vrabič Brodnjak and Muck^76^ and González et al.^77^. Hence, there exists a correlation between air permeability and porosity. The air permeability of paper possesses a significant role in determining its strength, visual aesthetics, and suitability for specific applications, such as packaging papers^61^. According to the findings presented in Table 6, the porosity of the 6.000 samples exhibited improvement when subjected to higher beating revolutions in comparison to the 4.000 samples. This suggests that the presence of nanocellulose likely served as a robust framework, effectively filling the voids between the larger fibers within the fiber network. It is plausible that the compressed nanocellulose facilitates the diffusion of air molecules by creating elongated channels. Consequently, this phenomenon is likely to impede the ease with which air volume can traverse through the paper’s structure^78^. The result was a paper with 5% NFC, 1.5% CS, and 6.000 beating revolutions averaging 9.30 mL/min. In comparison with the control sample, a substantial increment in porosity of 96.35% was observed. This improvement reflects a reduction in air permeability. According to the findings of Adnan et al.^79^, the incorporation of nanocellulose at a concentration of up to 10% resulted in a significant reduction of 50% in air permeability. The strong barrier qualities of nanocellulose, as demonstrated by El-Samahy et al.^80^, make it an interesting option for utilization in the papermaking sector, particularly in the realm of food packaging.

#### Antimicrobial properties

Several parameters influence the antibacterial activity of chitosan, for example, molecular weight**,** pH, chitosan derivatives, food components, type of microbe, deacetylation degree, and chitosan sources Riaz Rajoka et al.^81^. The antimicrobial outcomes presented in Table 7 demonstrate varying antibacterial reactions observed among the investigated substances. Different optical densities were observed when testing the antibacterial efficacy of samples containing 0% NFC, 5% NFC, and 5% NFC plus 1.5% chitosan at 6.000 revolutions. Then the optical densities (OD_600_) measurements were normalized against a blank sample.

**Table 7:** The antimicrobial activity of modified paper sheets in the presence of different microorganisms.

Microorganism	Gram Stain	Growth	Pulp fibers–6.000 Rev	5% NFC–6.000 Rev	5% NFC–1.5% CS–6.000 Rev	Reference Antibiotic
*Staphylococcus aureus*	Positive	0.298	0.289 (± 0.051)	0.240 (± 0.014)	0.207 (± 0.049)	0.201
*Escherichia coli*	Negative	0.491	0.470 (± 0.021)	0.445 (± 0.070)	0.410 (± 0.191)	0.280
*Salmonella choleraesuis*	Negative	0.500	0.490 (± 0.169)	0.460 (± 0.013)	0.437 (± 0.039)	0.300
*Candida albicans*	Yeast	0.304	0.300 (± 0.047)	0.242 (± 0.035)	0.203 (± 0.030)	0.170

Rev, Revolutions; NFC, Nanofibrillated cellulose; CS, Chitosan.

The highest level of antibacterial activity was seen in *Staphylococcus aureus*. At a rotational velocity of 6.000 revolutions per min, the sample consisting of 5% NFC and 1.5% CS exhibited the most potent antibacterial action against both gram-positive and gram-negative pathogens, including *Candida albicans*. The third specimen (OD_600_ = 0.207) displayed a lower pronounced microbial growth and close to the standard antibiotic (OD_600_ = 0.201). This observation suggests that the potency of the microbial growth is closely correlated with the concentration of chitosan present in the specimen. Furthermore, this particular specimen’s pH value of approximately 5.09 demonstrates the effectiveness of antimicrobial agents (chitosan) against *Staphylococcus aureus*. Based on a prior investigation conducted by Riaz Rajoka et al.^81^, it was shown that chitosan molecules with multiple positive charges (polycationic) establish interactions with membrane molecules with negative charges, including fatty acids, phospholipids, proteins, and polysaccharides. This phenomenon can be ascribed to the abundant presence of amino groups on the polymer’s surface. Furthermore, the study conducted by Hosseinnejad and Jafari^82^ revealed that chitosan with its derivatives possesses antimicrobial properties. This can be attributed to the presence of a positive charge on the + NH3 group of the glucosamine monomer at a pH level below 6.3. This positive charge allows it to interact with cells, disrupting their membranes and causing the release of their internal contents, which leads to cell death. In contrast, gram-negative bacteria exhibited lower resistance to these substances in comparison to conventional antibiotics targeting the two specific pathogens, *Escherichia coli* and *Salmonella choleraesuis*. Compared to specimens containing 0% NFC at 6.000 revolutions and 5% NFC at 6.000 revolutions, the microbial growth of yeast *Candida albicans* was much lower in specimens containing 5% NFC, 1.5% CS, and 6.000 revolutions (OD_600_ = 0.203), with OD_600_ values of 0.300 and 0.242, respectively. Additionally, it yielded a result that closely matched the standard antibiotic’s OD_600_ value of 0.201. Although the value of the standard antibiotic (OD_600_ 0.201) is higher, the observed value is lower. The findings of this study conform to the research conducted by El-Samahy et al.^80^, which demonstrated that the incorporation of nanocrystalline cellulose and chitosan into bleached bagasse pulp enhanced its antimicrobial properties. Consequently, the modified paper sheet exhibited increased antimicrobial activity. Furthermore, this reinforcement paper works in real food contact conditions or in packaging trials as follows: These antimicrobial papers have chitosan, which helps control gas exchange, slows down respiration, prevents mold growth, boosts the body’s defense mechanisms, and lowers ethylene production, making them capable of extending the freshness of perishable foods^83^.

## Conclusions

This work successfully developed a novel paper for food packaging applications by incorporating nanofibrillated cellulose and chitosan into an unbleached alkaline sulfite anthraquinone and methanol pulp suspension. The study’s findings reveal a statistically significant difference in the functional properties of modified paper when nanofibrillated cellulose and chitosan are added. Moreover, given the need to account for both economic and environmental factors in the papermaking process, this study opted for a composition consisting of 5% NFC, 1.5% CS, and 6.000 beating revolutions. Therefore, the addition of 5% NFC is deemed suitable due to the adverse effects of excessive NFC usage, such as drainage issues, increased energy requirements in plant operations, particularly in compression and drying procedures, and the exorbitant cost associated with it. This experiment resulted in the optimum values of a thickness of 13.71 μm, a tensile index of 85.16 Nm/g, and a burst index of 7.69 kPa m^2^/g. Besides that, it exhibited sufficient thermal stability, with the onset temperature of thermal degradation surpassing 220 °C. It is the highest temperature of tolerance for food packaging applications, resulting in 258.28 °C. Nevertheless, it yielded more significant outcomes in terms of barrier properties by enhancing smoothness by 11.93% and porosity by 96.35%. This reflects a decrease in air permeability of ASAM-reinforced paper. Likewise, the antimicrobial activity of the paper showed a notable improvement, with a 43.96% increase against *Staphylococcus aureus* and moderate increases of 14.42% and 19.76% against *Salmonella choleraesuis* and *Escherichia coli*, respectively. In the context of yeast, the prevalence of *Candida albicans* was determined to be 49.75%.

This study was subject to certain limitations, such as limited financial resources and time constraints. Future research must evaluate the morphological characteristics of the paper using a scanning electron microscope (SEM) to determine how NFC and chitosan are distributed among the fibers in the paper. Additionally, further investigation into barrier characteristics should be conducted by assessing the water vapor transmission rate (WVTR) characteristics of the sample. Furthermore, more future studies on antioxidant activity will know what kind of food could be stored in this application of packaging paper. In conclusion, the incorporation of 5% NFC and 1.5% chitosan at 6.000 beating revolutions significantly enhances the mechanical, thermal, barrier, and antimicrobial properties of ASAM unbleached paper; however, further research is needed to uncover its full potential.

## Data Availability

Data can be obtained on request from Corresponding author (M. Jawaid).
